# Triprolidinium dichloranilate–chloranilic acid–methanol–water (2/1/2/2)

**DOI:** 10.1107/S1600536812010136

**Published:** 2012-03-10

**Authors:** A. S. Dayananda, Ray J. Butcher, Mehmet Akkurt, H. S. Yathirajan, B. Narayana

**Affiliations:** a Department of Studies in Chemistry, University of Mysore, Manasagangotri, Mysore 570 006, India; b Department of Chemistry, Howard University, 525 College Street NW, Washington, DC 20059, USA; c Department of Physics, Faculty of Sciences, Erciyes University, 38039 Kayseri, Turkey; d Department of Studies in Chemistry, Mangalore University, Mangalagangotri 574 199, India

## Abstract

In the triprolidinium cation of the title compound {systematic name: 2-[1-(4-methyl­phen­yl)-3-(pyrrolidin-1-ium-1-yl)prop-1-en-1-yl]pyridin-1-ium bis­(2,5-dichloro-4-hy­droxy-3,6-dioxo­cyclo­hexa-1,4-dien-1-olate)–2,5-dichloro-3,6-dihy­droxy­cyclo­hexa-2,5-diene-1,4-dione–methanol–water (2/1/2/2)}, C_19_H_24_N_2_^2+^·2C_6_HCl_2_O_4_^−^·0.5C_6_H_2_Cl_2_O_4_·CH_3_OH·H_2_O, the N atoms on both the pyrrolidine and pyridine groups are protonated. The neutral chloranilic acid mol­ecule is on an inversion symmetry element and its hy­droxy H atoms are disordered over two positions with site-occupancy factors of 0.53 (6) and 0.47 (6). The methanol solvent mol­ecule is disordered over two positions in a 0.836 (4):0.164 (4) ratio. In the crystal, N—H⋯O, O—H⋯O and C—H⋯O inter­actions link the components. The crystal structure also features π–π inter­actions between the benzene rings [centroid–centroid distances = 3.5674 (15), 3.5225 (15) and 3.6347 (15) Å].

## Related literature

For the synthesis and spectroscopic studies of charge-transfer complexes between chloranilic acid and some heterocyclic amines in ethanol, see: Al-Attas *et al.* (2009 ▶). For spectroscopic studies of the inter­action between triprolidine hydro­chloride and serum albumins, see: Sandhya *et al.* (2011 ▶). For related structures, see: Adam *et al.* (2010 ▶); Dayananda *et al.* (2011 ▶); Dutkiewicz *et al.* (2010 ▶); Gotoh *et al.* (2010 ▶); Hakim Al-arique *et al.* (2010 ▶); Jasinski *et al.* (2010 ▶); Parvez & Sabir (1997 ▶); Udachin *et al.* (2011 ▶). For ring puckering parameters, see: Cremer & Pople (1975 ▶). 
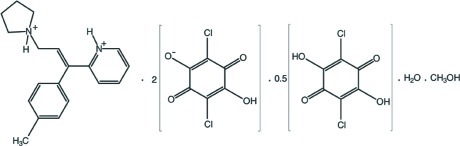


## Experimental

### 

#### Crystal data

C_19_H_24_N_2_^2+^·2C_6_HCl_2_O_4_^−^·0.5C_6_H_2_Cl_2_O_4_·CH_4_O·H_2_O
*M* *_r_* = 850.88
Monoclinic, 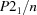

*a* = 9.1633 (2) Å
*b* = 32.3720 (7) Å
*c* = 12.9834 (4) Å
β = 106.685 (3)°
*V* = 3689.17 (17) Å^3^
*Z* = 4
Cu *K*α radiation
μ = 4.16 mm^−1^
*T* = 123 K
0.5 × 0.38 × 0.12 mm

#### Data collection

Agilent Xcalibur Ruby Gemini diffractometer
Absorption correction: multi-scan (*CrysAlis PRO*; Agilent, 2011 ▶) *T*_min_ = 0.188, *T*_max_ = 0.607
25133 measured reflections
7532 independent reflections
6721 reflections with *I* > 2σ(*I*)
*R*_int_ = 0.036

#### Refinement

*R*[*F* ^2^ > 2σ(*F* ^2^)] = 0.052
*wR*(*F* ^2^) = 0.137
*S* = 1.08
7532 reflections
510 parameters
4 restraints
H atoms treated by a mixture of independent and constrained refinement
Δρ_max_ = 1.08 e Å^−3^
Δρ_min_ = −0.29 e Å^−3^

### 

Data collection: *CrysAlis PRO* (Agilent, 2011 ▶); cell refinement: *CrysAlis PRO*; data reduction: *CrysAlis RED* (Agilent, 2011 ▶); program(s) used to solve structure: *SHELXS97* (Sheldrick, 2008 ▶); program(s) used to refine structure: *SHELXL97* (Sheldrick, 2008 ▶); molecular graphics: *ORTEP-3 for Windows* (Farrugia, 1997 ▶); software used to prepare material for publication: *WinGX* (Farrugia, 1999 ▶) and *PLATON* (Spek, 2009 ▶).

## Supplementary Material

Crystal structure: contains datablock(s) global, I. DOI: 10.1107/S1600536812010136/bt5841sup1.cif

Structure factors: contains datablock(s) I. DOI: 10.1107/S1600536812010136/bt5841Isup2.hkl

Supplementary material file. DOI: 10.1107/S1600536812010136/bt5841Isup3.cml

Additional supplementary materials:  crystallographic information; 3D view; checkCIF report

## Figures and Tables

**Table 1:** Hydrogen-bond geometry (Å, °)

*D*—H⋯*A*	*D*—H	H⋯*A*	*D*⋯*A*	*D*—H⋯*A*
O4*A*—H4*AA*⋯O3*A*	0.84	2.15	2.629 (3)	116
O4*A*—H4*AA*⋯O1*W*^i^	0.84	1.92	2.672 (3)	148
N1—H1*C*⋯O3*A*	0.93	1.78	2.699 (3)	167
O2*B*—H2*BA*⋯O1*B*	0.84	2.19	2.655 (3)	115
O2*B*—H2*BA*⋯O1*A*^ii^	0.84	2.50	3.012 (3)	121
O2*B*—H2*BA*⋯O2*A*^ii^	0.84	2.08	2.776 (3)	139
N2—H2*C*⋯O3*B*	0.88	2.55	2.900 (3)	104
N2—H2*C*⋯O4*B*	0.88	1.79	2.667 (3)	175
O2*C*—H2*CA*⋯O1*C*^iii^	0.84	2.21	2.680 (4)	116
O1*W*—H1*W*1⋯O1*S*	0.90 (3)	2.06 (3)	2.882 (3)	152 (4)
O1*W*—H1*W*2⋯O2*B*	0.82 (5)	2.18 (4)	2.976 (3)	162 (4)
C1—H1*B*⋯O1*A*^iv^	0.99	2.34	3.286 (5)	160
C3—H3*A*⋯O1*B*^v^	0.99	2.47	3.138 (5)	124
C4—H4*B*⋯O2*A*^vi^	0.99	2.37	3.229 (3)	144
C4—H4*B*⋯O1*B*^v^	0.99	2.34	2.994 (3)	123
C5—H5*B*⋯O1*B*^v^	0.99	2.44	3.186 (3)	132
C9—H9*A*⋯Cl2*A*^vi^	0.95	2.82	3.525 (3)	131
C9—H9*A*⋯O2*A*^vi^	0.95	2.46	3.363 (3)	158
C18—H18*A*⋯Cl2*B*^i^	0.95	2.68	3.467 (3)	140
C19—H19*B*⋯O1*A*^ii^	0.98	2.55	3.102 (4)	116

Symmetry codes: (i) 
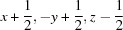
; (ii) 
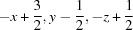
; (iii) 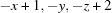; (iv) 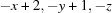; (v) 
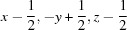
; (vi) 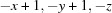.

**Fig. 1.**
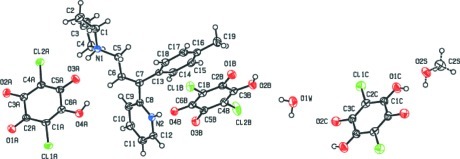
Molecular structure of (I) with the atom labeling scheme. Displacement ellipsoids for non-H atoms are drawn at the 50% probability level. Only the major component of the disorder is shown.

**Fig. 2.**
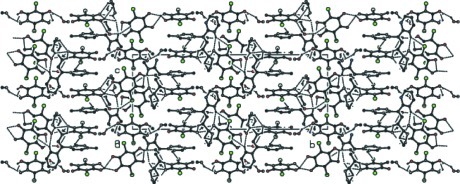
Perspective view of the crystal packing and hydrogen bonding of (I) down the a axis. For clarity, hydrogen atoms not involved in hydrogen bonding have been omitted and only the major component of the disorder is shown.

## supplementary crystallographic information

### Comment

Triprolidine (Systematic name: 2-[(*E*)-1-(4-methylphenyl)-3-pyrrolidin-1-yl-prop- 1-enyl]pyridine) is an over-the-counter antihistamine with anticholinergic properties. It is used to combat the symptoms associated with allergies and is sometimes combined with other cold medications designed to provide general relief for flu-like symptoms. Like many over-the-counter antihistamines, the most common side effect is drowsiness. Triprolidine is a quick acting drug that can clear congestion and stop runny noses in 15–30 minutes. The interaction between triprolidine hydrochloride (TRP) and serum albumins *viz*. bovine serum albumin (BSA) and human serum albumin (HSA) has been studied by spectroscopic methods (Sandhya *et al.*, 2011).

Chloranilic acid is a strong dibasic organic acid which exhibits electron-acceptor properties on one hand and acidic properties leading to formation of hydrogen bonds on the other hand. In the case of stronger bases the proton-transfer, hydrogen bonded ion pairs will be formed which is interesting from the point of view of electron transfer reactions in biological systems. Also, protonation of the donor from acidic acceptors are generally a route for the formation of ion pair adducts. The synthesis and spectroscopic studies of charge transfer complexes between chloranilic acid and some heterocyclic amines in ethanol (Al-Attas, Habeeb & Al-Raimi, 2009) have been studied. The crystal structures of triprolidine tetrachlorocuprate (II) (Parvez & Sabir, 1997), triethylammonium hydrogen chloranilate (Gotoh *et al.*, 2010), chloranilic acid: a redetermination at 100 K (Dutkiewicz *et al.*, 2010), bis(3-picoline) chloranilate chloranilic acid (Adam *et al.*, 2010), Gabapentin-lactum-chloranilic acid (Jasinski *et al.*, 2010), bis(2-{[3-methyl-4-(2,2,2-trifluoroethoxy)-2-pyridyl]methylsulfanyl}-1H,3*H*-benzimidazolium) 2,5-dichloro-3,6-dioxocyclohexa-1,4-diene-1,4-diolate (Hakim Al-arique *et al.*, 2010), bis(guanidinium) chloranilate (Udachin *et al.*, 2011) and triprolinidium dipicrate (Dayananda *et al.*, 2011) have been reported. In view of the importance of triprolidine, the paper reports the crystal structure of the title compound, (I).

As shown in Fig. 1, in the triprolidinium cation of (I), the N atoms on the pyridinium and pyrrolidine groups are protonated. The pyrrolidine group has an envelope conformation [the puckering parameters (Cremer & Pople, 1975) are Q(2) = 0.378 (4) Å, φ(2) = 1252.8 (5)°].

The crystal structure is stabilized by N—H···O, O—H···O and C—H···O interactions (Table 1, Fig. 2). Furthermore, the crystal structure is stabilized *via*π-π interactions between the benzene rings [*Cg*3···*Cg*5(*x*, *y*, *z*) = 3.5674 (15) Å, *Cg*3···*Cg*5(-1/2 + *x*, 1/2 - *y*, -1/2 + *z*) = 3.5225 (15) Å, *Cg*4···*Cg*4(2 - *x*, 1 - *y*, -*z*) = 3.6347 (15) Å; where *Cg*3, *Cg*4 and g5 are the centroids of the C13—C18, C1A–C6A and C1B–C6B benzene rings, respectively].

### Experimental

Triprolidine hydrochloride (3.148 g, 0.01 mol) in 10 ml of methanol was mixed with chloranilic acid (2.09 g, 0.01 mol) in 10 ml of methanol. The mixture was kept aside for three days at room temperature. The formed salt was filtered and dried in a vacuum desiccator over phosphorous pentoxide. The compound was recrystallized from methanol solution by slow evaporation (m.p.: 448–450 K with charring).

### Refinement

The atoms of the methanol solvent molecule are disordered over two positions with the site-occupancy factors of 0.836 (4) and 0.164 (4). The hydroxyl H atoms of the neutral chloranilate molecule lying on an inversion centre are disordered over two positions with the site-occupancy factors of 0.53 (6) and 0.47 (6). The water H atoms were located in a difference Fourier map and refined with *U*_iso_(H) = 1.5*U*_eq_(O) and using the *DFIX* restraints for the O—H bond of 0.82 Å and the H···H distance of 1.297 Å. All of the remaining H atoms were placed in their calculated positions and refined using the riding model with C–H lengths of 0.84 Å (OH), 0.88 Å (NH), 0.93 Å (NH), 0.95 Å (CH), 0.99 Å (CH_2_) or 0.98 Å (CH_3_). Their isotropic displacement parameters were set to 1.2 (NH, CH, CH_2_) or 1.5 (OH, CH_3_) times *U*_eq_ of the parent atom.

### Crystal data

**Table:**

C_19_H_24_N_2_^2+^·2C_6_HCl_2_O_4_^−^·0.5C_6_H_2_Cl_2_O_4_·CH_4_O·H_2_O	*F*(000) = 1752
*M**_r_* = 850.88	*D*_x_ = 1.532 Mg m^−^^3^
Monoclinic, *P*2_1_/*n*	Cu *K*α radiation, λ = 1.54184 Å
Hall symbol: -P 2yn	Cell parameters from 10725 reflections
*a* = 9.1633 (2) Å	θ = 2.7–75.5°
*b* = 32.3720 (7) Å	µ = 4.16 mm^−^^1^
*c* = 12.9834 (4) Å	*T* = 123 K
β = 106.685 (3)°	Plate, dark brown
*V* = 3689.17 (17) Å^3^	0.5 × 0.38 × 0.12 mm
*Z* = 4	

### Data collection

**Table:**

Agilent Xcalibur Ruby Gemini diffractometer	7532 independent reflections
Radiation source: Enhance (Cu) X-ray Source	6721 reflections with *I* > 2σ(*I*)
Graphite monochromator	*R*_int_ = 0.036
Detector resolution: 10.5081 pixels mm^-1^	θ_max_ = 75.7°, θ_min_ = 2.7°
ω scans	*h* = −9→11
Absorption correction: multi-scan (*CrysAlis PRO*; Agilent, 2011)	*k* = −39→40
*T*_min_ = 0.188, *T*_max_ = 0.607	*l* = −16→16
25133 measured reflections	

### Refinement

**Table:**

Refinement on *F*^2^	Primary atom site location: structure-invariant direct methods
Least-squares matrix: full	Secondary atom site location: difference Fourier map
*R*[*F*^2^ > 2σ(*F*^2^)] = 0.052	Hydrogen site location: inferred from neighbouring sites
*wR*(*F*^2^) = 0.137	H atoms treated by a mixture of independent and constrained refinement
*S* = 1.08	*w* = 1/[σ^2^(*F*_o_^2^) + (0.065*P*)^2^ + 4.4856*P*] where *P* = (*F*_o_^2^ + 2*F*_c_^2^)/3
7532 reflections	(Δ/σ)_max_ = 0.001
510 parameters	Δρ_max_ = 1.08 e Å^−^^3^
4 restraints	Δρ_min_ = −0.29 e Å^−^^3^

### Special details

**Table:**

Geometry. Bond distances, angles *etc*. have been calculated using the rounded fractional coordinates. All su's are estimated from the variances of the (full) variance-covariance matrix. The cell e.s.d.'s are taken into account in the estimation of distances, angles and torsion angles
Refinement. Refinement on *F*^2^ for ALL reflections except those flagged by the user for potential systematic errors. Weighted *R*-factors *wR* and all goodnesses of fit *S* are based on *F*^2^, conventional *R*-factors *R* are based on *F*, with *F* set to zero for negative *F*^2^. The observed criterion of *F*^2^ > σ(*F*^2^) is used only for calculating -*R*-factor-obs *etc*. and is not relevant to the choice of reflections for refinement. *R*-factors based on *F*^2^ are statistically about twice as large as those based on *F*, and *R*-factors based on ALL data will be even larger.

### Fractional atomic coordinates and isotropic or equivalent isotropic displacement parameters (Å^2^)

**Table:**

	*x*	*y*	*z*	*U*_iso_*/*U*_eq_	Occ. (<1)
N1	0.6083 (3)	0.38941 (7)	−0.02161 (17)	0.0253 (6)	
N2	0.5846 (2)	0.37690 (6)	0.42666 (17)	0.0219 (6)	
C1	0.6360 (4)	0.36770 (10)	−0.1171 (2)	0.0396 (10)	
C2	0.5318 (5)	0.38991 (13)	−0.2126 (3)	0.0566 (13)	
C3	0.3923 (4)	0.39853 (14)	−0.1786 (3)	0.0562 (13)	
C4	0.4523 (3)	0.40954 (9)	−0.0600 (2)	0.0329 (8)	
C5	0.6297 (3)	0.36064 (8)	0.0717 (2)	0.0256 (7)	
C6	0.5944 (3)	0.38106 (8)	0.1656 (2)	0.0262 (7)	
C7	0.5387 (3)	0.36176 (8)	0.2373 (2)	0.0237 (7)	
C8	0.5085 (3)	0.38700 (7)	0.3248 (2)	0.0229 (7)	
C9	0.4040 (3)	0.41905 (8)	0.3069 (2)	0.0271 (7)	
C10	0.3852 (3)	0.44096 (8)	0.3946 (2)	0.0300 (8)	
C11	0.4688 (3)	0.43054 (8)	0.4977 (2)	0.0279 (8)	
C12	0.5685 (3)	0.39775 (8)	0.5118 (2)	0.0243 (7)	
C13	0.5056 (3)	0.31700 (8)	0.23996 (19)	0.0240 (7)	
C14	0.3774 (3)	0.30343 (9)	0.2686 (2)	0.0302 (8)	
C15	0.3474 (4)	0.26157 (10)	0.2725 (2)	0.0375 (9)	
C16	0.4406 (4)	0.23209 (9)	0.2476 (2)	0.0376 (9)	
C17	0.5687 (4)	0.24541 (9)	0.2205 (2)	0.0346 (8)	
C18	0.6013 (3)	0.28713 (8)	0.2174 (2)	0.0292 (8)	
C19	0.4090 (5)	0.18638 (10)	0.2523 (3)	0.0536 (13)	
Cl1A	1.17727 (7)	0.54478 (2)	0.31560 (5)	0.0301 (2)	
Cl2A	0.65597 (7)	0.50251 (2)	−0.11080 (5)	0.0304 (2)	
O1A	1.0309 (3)	0.60103 (6)	0.13472 (17)	0.0373 (6)	
O2A	0.8198 (2)	0.58421 (6)	−0.05149 (15)	0.0294 (5)	
O3A	0.8213 (2)	0.44654 (6)	0.06840 (16)	0.0326 (6)	
O4A	1.0396 (2)	0.46331 (6)	0.24478 (15)	0.0292 (5)	
C1A	1.0377 (3)	0.53290 (8)	0.1979 (2)	0.0244 (7)	
C2A	0.9846 (3)	0.56589 (8)	0.1210 (2)	0.0240 (7)	
C3A	0.8602 (3)	0.55558 (8)	0.0146 (2)	0.0239 (7)	
C4A	0.8028 (3)	0.51523 (8)	0.00329 (19)	0.0235 (7)	
C5A	0.8605 (3)	0.48339 (8)	0.0769 (2)	0.0236 (7)	
C6A	0.9862 (3)	0.49402 (8)	0.1777 (2)	0.0234 (7)	
Cl1B	0.95280 (7)	0.24300 (2)	0.41301 (6)	0.0322 (2)	
Cl2B	0.38936 (10)	0.23353 (2)	0.60046 (9)	0.0526 (3)	
O1B	0.7894 (2)	0.16893 (6)	0.45954 (17)	0.0319 (6)	
O2B	0.5596 (2)	0.16314 (6)	0.54515 (17)	0.0314 (6)	
O3B	0.5501 (2)	0.30750 (6)	0.55902 (17)	0.0323 (6)	
O4B	0.7820 (2)	0.31432 (5)	0.47158 (15)	0.0257 (5)	
C1B	0.7997 (3)	0.24176 (8)	0.46650 (19)	0.0229 (7)	
C2B	0.7414 (3)	0.20213 (8)	0.48159 (19)	0.0236 (7)	
C3B	0.6074 (3)	0.20037 (8)	0.5271 (2)	0.0252 (7)	
C4B	0.5425 (3)	0.23515 (8)	0.5487 (2)	0.0275 (7)	
C5B	0.6020 (3)	0.27584 (8)	0.5340 (2)	0.0244 (7)	
C6B	0.7379 (3)	0.27837 (8)	0.48720 (19)	0.0227 (7)	
Cl1C	0.18813 (8)	0.00452 (2)	0.81624 (6)	0.0405 (2)	
O1C	0.3493 (3)	−0.07006 (7)	0.92826 (19)	0.0422 (7)	
O2C	0.3816 (3)	0.07432 (6)	0.92023 (18)	0.0380 (6)	
C1C	0.4153 (3)	−0.03683 (8)	0.9587 (2)	0.0279 (8)	
C2C	0.3582 (3)	0.00227 (9)	0.9176 (2)	0.0287 (7)	
C3C	0.4335 (3)	0.03859 (8)	0.9551 (2)	0.0275 (7)	
O1S	0.5299 (3)	0.14776 (7)	0.9411 (2)	0.0366 (8)	0.836 (4)
C1S	0.6758 (5)	0.16296 (13)	1.0010 (4)	0.0446 (14)	0.836 (4)
O2S	0.4348 (14)	−0.1470 (4)	0.9421 (12)	0.0366 (8)	0.164 (4)
C2S	0.298 (2)	−0.1688 (7)	0.940 (2)	0.0446 (14)	0.164 (4)
O1W	0.4911 (3)	0.11809 (7)	0.7256 (2)	0.0500 (8)	
H1A	0.60940	0.33800	−0.11770	0.0480*	
H1B	0.74380	0.37030	−0.11670	0.0480*	
H1C	0.68020	0.41040	−0.00100	0.0300*	
H2A	0.57880	0.41590	−0.22770	0.0680*	
H2B	0.50750	0.37220	−0.27750	0.0680*	
H2C	0.64740	0.35570	0.43770	0.0260*	
H3A	0.32570	0.37390	−0.18890	0.0670*	
H3B	0.33380	0.42180	−0.22020	0.0670*	
H4A	0.46070	0.43990	−0.05030	0.0390*	
H4B	0.38390	0.39870	−0.01960	0.0390*	
H5A	0.73630	0.35070	0.09410	0.0310*	
H5B	0.56230	0.33640	0.04910	0.0310*	
H6A	0.61320	0.40990	0.17490	0.0310*	
H9A	0.34600	0.42600	0.23580	0.0330*	
H10A	0.31460	0.46310	0.38340	0.0360*	
H11A	0.45790	0.44570	0.55770	0.0330*	
H12A	0.62610	0.38990	0.58230	0.0290*	
H14A	0.31060	0.32300	0.28540	0.0360*	
H15A	0.26040	0.25300	0.29280	0.0450*	
H17A	0.63510	0.22560	0.20370	0.0410*	
H18A	0.69050	0.29550	0.19960	0.0350*	
H19A	0.33110	0.18210	0.28950	0.0800*	
H19B	0.50290	0.17210	0.29140	0.0800*	
H19C	0.37270	0.17530	0.17910	0.0800*	
H4AA	0.99890	0.44110	0.21770	0.0350*	
H2BA	0.60650	0.14510	0.52060	0.0380*	
H2CA	0.44580	0.09250	0.94910	0.0460*	0.53 (6)
H1CA	0.40330	−0.08970	0.96060	0.0510*	0.47 (6)
H1S	0.46160	0.16340	0.95050	0.0550*	0.836 (4)
H1S1	0.75540	0.14480	0.98980	0.0670*	0.836 (4)
H1S2	0.68210	0.16350	1.07760	0.0670*	0.836 (4)
H1S3	0.68990	0.19100	0.97680	0.0670*	0.836 (4)
H2S	0.46300	−0.13290	0.99860	0.0550*	0.164 (4)
H2S1	0.21600	−0.14900	0.93630	0.0670*	0.164 (4)
H2S2	0.31540	−0.18550	1.00540	0.0670*	0.164 (4)
H2S3	0.26970	−0.18690	0.87690	0.0670*	0.164 (4)
H1W1	0.509 (6)	0.1352 (12)	0.782 (2)	0.0750*	
H1W2	0.526 (6)	0.1329 (12)	0.687 (3)	0.0750*	

### Atomic displacement parameters (Å^2^)

**Table:**

	*U*^11^	*U*^22^	*U*^33^	*U*^12^	*U*^13^	*U*^23^
N1	0.0281 (11)	0.0271 (10)	0.0237 (10)	−0.0086 (8)	0.0121 (9)	−0.0049 (8)
N2	0.0219 (10)	0.0215 (9)	0.0227 (10)	0.0015 (7)	0.0073 (8)	0.0007 (8)
C1	0.0541 (19)	0.0407 (16)	0.0315 (15)	−0.0100 (14)	0.0243 (14)	−0.0120 (12)
C2	0.078 (3)	0.066 (2)	0.0261 (16)	−0.031 (2)	0.0154 (16)	−0.0012 (15)
C3	0.050 (2)	0.077 (3)	0.0341 (18)	−0.0118 (18)	−0.0001 (15)	0.0200 (17)
C4	0.0323 (14)	0.0280 (13)	0.0394 (15)	−0.0021 (11)	0.0121 (12)	0.0073 (11)
C5	0.0275 (12)	0.0261 (12)	0.0244 (12)	−0.0015 (9)	0.0096 (10)	−0.0012 (9)
C6	0.0305 (13)	0.0247 (12)	0.0245 (12)	−0.0020 (10)	0.0096 (10)	−0.0016 (9)
C7	0.0236 (12)	0.0261 (12)	0.0203 (11)	0.0005 (9)	0.0044 (9)	−0.0019 (9)
C8	0.0239 (12)	0.0241 (11)	0.0215 (12)	−0.0028 (9)	0.0079 (9)	−0.0002 (9)
C9	0.0294 (13)	0.0285 (12)	0.0215 (12)	0.0024 (10)	0.0043 (10)	0.0027 (9)
C10	0.0307 (13)	0.0274 (13)	0.0319 (14)	0.0058 (10)	0.0088 (11)	−0.0001 (10)
C11	0.0284 (13)	0.0302 (13)	0.0265 (13)	−0.0008 (10)	0.0102 (10)	−0.0052 (10)
C12	0.0265 (12)	0.0269 (12)	0.0182 (11)	−0.0021 (9)	0.0043 (9)	−0.0017 (9)
C13	0.0279 (12)	0.0268 (12)	0.0147 (11)	−0.0028 (9)	0.0021 (9)	−0.0002 (9)
C14	0.0309 (13)	0.0359 (14)	0.0217 (12)	−0.0048 (11)	0.0041 (10)	−0.0003 (10)
C15	0.0396 (16)	0.0459 (17)	0.0222 (13)	−0.0172 (13)	0.0013 (11)	0.0051 (11)
C16	0.0557 (19)	0.0297 (14)	0.0180 (12)	−0.0095 (12)	−0.0043 (12)	0.0039 (10)
C17	0.0477 (17)	0.0276 (13)	0.0224 (13)	0.0048 (12)	0.0004 (12)	0.0006 (10)
C18	0.0348 (14)	0.0299 (13)	0.0221 (12)	0.0011 (10)	0.0069 (10)	0.0001 (10)
C19	0.077 (3)	0.0346 (17)	0.0345 (17)	−0.0173 (16)	−0.0076 (16)	0.0073 (13)
Cl1A	0.0304 (3)	0.0339 (3)	0.0225 (3)	−0.0066 (2)	0.0019 (2)	−0.0053 (2)
Cl2A	0.0264 (3)	0.0360 (3)	0.0240 (3)	−0.0046 (2)	−0.0004 (2)	0.0010 (2)
O1A	0.0485 (12)	0.0256 (10)	0.0335 (11)	−0.0067 (8)	0.0051 (9)	−0.0019 (8)
O2A	0.0291 (9)	0.0286 (9)	0.0290 (9)	0.0004 (7)	0.0062 (8)	0.0045 (7)
O3A	0.0330 (10)	0.0255 (9)	0.0342 (10)	−0.0055 (7)	0.0013 (8)	−0.0004 (8)
O4A	0.0310 (10)	0.0265 (9)	0.0253 (9)	−0.0024 (7)	0.0006 (7)	0.0017 (7)
C1A	0.0224 (11)	0.0307 (13)	0.0183 (11)	−0.0014 (9)	0.0031 (9)	−0.0041 (9)
C2A	0.0250 (12)	0.0250 (12)	0.0231 (12)	−0.0016 (9)	0.0088 (10)	−0.0040 (9)
C3A	0.0216 (11)	0.0280 (12)	0.0239 (12)	0.0025 (9)	0.0092 (9)	0.0014 (9)
C4A	0.0190 (11)	0.0301 (12)	0.0198 (11)	−0.0011 (9)	0.0028 (9)	−0.0026 (9)
C5A	0.0218 (11)	0.0265 (12)	0.0230 (12)	−0.0026 (9)	0.0072 (9)	−0.0036 (9)
C6A	0.0219 (11)	0.0284 (12)	0.0199 (11)	0.0007 (9)	0.0062 (9)	0.0003 (9)
Cl1B	0.0295 (3)	0.0312 (3)	0.0419 (4)	0.0047 (2)	0.0199 (3)	0.0024 (3)
Cl2B	0.0561 (5)	0.0320 (4)	0.0915 (7)	0.0010 (3)	0.0562 (5)	0.0018 (4)
O1B	0.0337 (10)	0.0257 (9)	0.0384 (11)	0.0038 (7)	0.0135 (8)	−0.0029 (8)
O2B	0.0343 (10)	0.0238 (9)	0.0406 (11)	−0.0008 (7)	0.0181 (9)	−0.0023 (8)
O3B	0.0374 (10)	0.0271 (9)	0.0386 (11)	0.0042 (8)	0.0209 (9)	0.0011 (8)
O4B	0.0252 (9)	0.0242 (9)	0.0280 (9)	0.0027 (7)	0.0083 (7)	0.0028 (7)
C1B	0.0190 (11)	0.0303 (13)	0.0198 (11)	0.0030 (9)	0.0060 (9)	0.0008 (9)
C2B	0.0217 (11)	0.0278 (12)	0.0191 (11)	0.0050 (9)	0.0025 (9)	−0.0003 (9)
C3B	0.0274 (12)	0.0262 (12)	0.0203 (12)	0.0001 (9)	0.0043 (10)	0.0013 (9)
C4B	0.0256 (12)	0.0321 (13)	0.0278 (13)	0.0007 (10)	0.0125 (10)	0.0010 (10)
C5B	0.0260 (12)	0.0267 (12)	0.0201 (11)	0.0043 (9)	0.0061 (10)	0.0022 (9)
C6B	0.0209 (11)	0.0274 (12)	0.0178 (11)	0.0018 (9)	0.0025 (9)	0.0021 (9)
Cl1C	0.0273 (3)	0.0485 (4)	0.0392 (4)	−0.0011 (3)	−0.0006 (3)	0.0051 (3)
O1C	0.0395 (12)	0.0353 (11)	0.0474 (13)	−0.0103 (9)	0.0055 (10)	0.0020 (9)
O2C	0.0399 (11)	0.0314 (10)	0.0379 (11)	0.0089 (8)	0.0037 (9)	0.0012 (8)
C1C	0.0278 (13)	0.0277 (13)	0.0304 (13)	−0.0035 (10)	0.0118 (11)	−0.0012 (10)
C2C	0.0196 (11)	0.0412 (15)	0.0248 (12)	0.0004 (10)	0.0057 (10)	0.0015 (10)
C3C	0.0291 (13)	0.0266 (12)	0.0296 (13)	0.0037 (10)	0.0131 (11)	0.0040 (10)
O1S	0.0376 (13)	0.0251 (11)	0.0524 (15)	−0.0004 (9)	0.0216 (11)	−0.0056 (10)
C1S	0.037 (2)	0.041 (2)	0.051 (3)	0.0043 (15)	0.0052 (19)	−0.0026 (19)
O2S	0.0376 (13)	0.0251 (11)	0.0524 (15)	−0.0004 (9)	0.0216 (11)	−0.0056 (10)
C2S	0.037 (2)	0.041 (2)	0.051 (3)	0.0043 (15)	0.0052 (19)	−0.0026 (19)
O1W	0.0708 (17)	0.0330 (11)	0.0515 (15)	−0.0025 (11)	0.0260 (13)	0.0042 (10)

### Geometric parameters (Å, º)

**Table:**

Cl1A—C1A	1.731 (3)	C16—C17	1.387 (5)
Cl2A—C4A	1.741 (3)	C16—C19	1.512 (4)
Cl1B—C1B	1.735 (3)	C17—C18	1.386 (4)
Cl2B—C4B	1.722 (3)	C1—H1A	0.9900
Cl1C—C2C	1.729 (3)	C1—H1B	0.9900
O1A—C2A	1.209 (3)	C2—H2A	0.9900
O2A—C3A	1.245 (3)	C2—H2B	0.9900
O3A—C5A	1.242 (3)	C3—H3A	0.9900
O4A—C6A	1.320 (3)	C3—H3B	0.9900
O4A—H4AA	0.8400	C4—H4A	0.9900
O1B—C2B	1.226 (3)	C4—H4B	0.9900
O2B—C3B	1.326 (3)	C5—H5A	0.9900
O3B—C5B	1.213 (3)	C5—H5B	0.9900
O4B—C6B	1.267 (3)	C6—H6A	0.9500
O2B—H2BA	0.8400	C9—H9A	0.9500
O1C—C1C	1.242 (4)	C10—H10A	0.9500
O2C—C3C	1.283 (3)	C11—H11A	0.9500
O1C—H1CA	0.8400	C12—H12A	0.9500
O2C—H2CA	0.8400	C14—H14A	0.9500
O1S—C1S	1.427 (6)	C15—H15A	0.9500
O1S—H1S	0.8400	C17—H17A	0.9500
N1—C5	1.496 (3)	C18—H18A	0.9500
N1—C4	1.519 (4)	C19—H19A	0.9800
N1—C1	1.509 (4)	C19—H19C	0.9800
N2—C8	1.346 (3)	C19—H19B	0.9800
N2—C12	1.339 (3)	C1A—C6A	1.343 (4)
O2S—C2S	1.43 (2)	C1A—C2A	1.448 (4)
N1—H1C	0.9300	C2A—C3A	1.554 (4)
N2—H2C	0.8800	C3A—C4A	1.400 (4)
O2S—H2S	0.8400	C4A—C5A	1.402 (4)
O1W—H1W2	0.82 (5)	C5A—C6A	1.514 (4)
O1W—H1W1	0.90 (3)	C1B—C6B	1.373 (4)
C1—C2	1.512 (5)	C1B—C2B	1.425 (4)
C2—C3	1.494 (6)	C2B—C3B	1.509 (4)
C3—C4	1.521 (5)	C3B—C4B	1.340 (4)
C5—C6	1.501 (4)	C4B—C5B	1.459 (4)
C6—C7	1.338 (4)	C5B—C6B	1.537 (4)
C7—C13	1.483 (4)	C1C—C3C^i^	1.512 (4)
C7—C8	1.488 (4)	C1C—C2C	1.414 (4)
C8—C9	1.386 (4)	C2C—C3C	1.379 (4)
C9—C10	1.394 (4)	C1S—H1S1	0.9800
C10—C11	1.380 (4)	C1S—H1S2	0.9800
C11—C12	1.378 (4)	C1S—H1S3	0.9800
C13—C18	1.392 (4)	C2S—H2S3	0.9800
C13—C14	1.401 (4)	C2S—H2S1	0.9800
C14—C15	1.387 (4)	C2S—H2S2	0.9800
C15—C16	1.380 (5)		
			
C6A—O4A—H4AA	109.00	C10—C11—H11A	121.00
C3B—O2B—H2BA	109.00	C11—C12—H12A	120.00
C1C—O1C—H1CA	109.00	N2—C12—H12A	120.00
C3C—O2C—H2CA	109.00	C13—C14—H14A	120.00
C1S—O1S—H1S	109.00	C15—C14—H14A	120.00
C4—N1—C5	115.2 (2)	C16—C15—H15A	119.00
C1—N1—C4	107.1 (2)	C14—C15—H15A	119.00
C1—N1—C5	111.2 (2)	C18—C17—H17A	120.00
C8—N2—C12	122.7 (2)	C16—C17—H17A	119.00
C5—N1—H1C	108.00	C17—C18—H18A	119.00
C4—N1—H1C	108.00	C13—C18—H18A	119.00
C1—N1—H1C	108.00	H19A—C19—H19C	109.00
C12—N2—H2C	119.00	C16—C19—H19B	109.00
C8—N2—H2C	119.00	C16—C19—H19C	110.00
C2S—O2S—H2S	109.00	H19A—C19—H19B	109.00
H1W1—O1W—H1W2	97 (4)	H19B—C19—H19C	109.00
N1—C1—C2	103.7 (3)	C16—C19—H19A	109.00
C1—C2—C3	103.8 (3)	Cl1A—C1A—C2A	117.51 (19)
C2—C3—C4	104.6 (3)	C2A—C1A—C6A	121.8 (2)
N1—C4—C3	105.3 (2)	Cl1A—C1A—C6A	120.6 (2)
N1—C5—C6	112.0 (2)	C1A—C2A—C3A	118.1 (2)
C5—C6—C7	125.0 (2)	O1A—C2A—C3A	118.0 (2)
C6—C7—C13	126.4 (2)	O1A—C2A—C1A	123.9 (2)
C8—C7—C13	115.8 (2)	O2A—C3A—C4A	126.4 (2)
C6—C7—C8	117.8 (2)	C2A—C3A—C4A	116.8 (2)
N2—C8—C9	119.0 (2)	O2A—C3A—C2A	116.7 (2)
C7—C8—C9	123.7 (2)	Cl2A—C4A—C3A	119.03 (19)
N2—C8—C7	117.3 (2)	C3A—C4A—C5A	123.9 (2)
C8—C9—C10	119.1 (2)	Cl2A—C4A—C5A	117.0 (2)
C9—C10—C11	120.3 (2)	O3A—C5A—C6A	115.1 (2)
C10—C11—C12	118.7 (2)	C4A—C5A—C6A	117.8 (2)
N2—C12—C11	120.3 (2)	O3A—C5A—C4A	127.1 (2)
C7—C13—C14	120.4 (2)	O4A—C6A—C5A	116.4 (2)
C7—C13—C18	121.9 (2)	C1A—C6A—C5A	121.3 (2)
C14—C13—C18	117.7 (2)	O4A—C6A—C1A	122.3 (2)
C13—C14—C15	120.4 (3)	C2B—C1B—C6B	123.9 (3)
C14—C15—C16	121.7 (3)	Cl1B—C1B—C6B	119.0 (2)
C15—C16—C19	121.9 (3)	Cl1B—C1B—C2B	117.0 (2)
C17—C16—C19	120.0 (3)	O1B—C2B—C3B	116.4 (2)
C15—C16—C17	118.1 (3)	C1B—C2B—C3B	117.9 (2)
C16—C17—C18	121.0 (3)	O1B—C2B—C1B	125.7 (3)
C13—C18—C17	121.1 (3)	C2B—C3B—C4B	120.7 (2)
N1—C1—H1A	111.00	O2B—C3B—C4B	122.5 (3)
C2—C1—H1A	111.00	O2B—C3B—C2B	116.8 (2)
H1A—C1—H1B	109.00	Cl2B—C4B—C3B	121.1 (2)
N1—C1—H1B	111.00	Cl2B—C4B—C5B	117.1 (2)
C2—C1—H1B	111.00	C3B—C4B—C5B	121.8 (3)
C3—C2—H2A	111.00	C4B—C5B—C6B	118.4 (2)
C1—C2—H2B	111.00	O3B—C5B—C6B	119.1 (2)
C1—C2—H2A	111.00	O3B—C5B—C4B	122.5 (3)
H2A—C2—H2B	109.00	C1B—C6B—C5B	117.3 (2)
C3—C2—H2B	111.00	O4B—C6B—C5B	116.4 (2)
C4—C3—H3B	111.00	O4B—C6B—C1B	126.4 (3)
H3A—C3—H3B	109.00	O1C—C1C—C2C	124.2 (3)
C2—C3—H3A	111.00	C2C—C1C—C3C^i^	118.3 (2)
C4—C3—H3A	111.00	O1C—C1C—C3C^i^	117.5 (2)
C2—C3—H3B	111.00	C1C—C2C—C3C	122.4 (2)
C3—C4—H4B	111.00	Cl1C—C2C—C3C	118.9 (2)
C3—C4—H4A	111.00	Cl1C—C2C—C1C	118.7 (2)
N1—C4—H4A	111.00	C1C^i^—C3C—C2C	119.2 (2)
H4A—C4—H4B	109.00	O2C—C3C—C2C	123.2 (3)
N1—C4—H4B	111.00	O2C—C3C—C1C^i^	117.6 (2)
H5A—C5—H5B	108.00	O1S—C1S—H1S3	109.00
C6—C5—H5A	109.00	O1S—C1S—H1S1	109.00
N1—C5—H5B	109.00	O1S—C1S—H1S2	109.00
C6—C5—H5B	109.00	H1S2—C1S—H1S3	109.00
N1—C5—H5A	109.00	H1S1—C1S—H1S2	109.00
C7—C6—H6A	117.00	H1S1—C1S—H1S3	110.00
C5—C6—H6A	118.00	O2S—C2S—H2S2	109.00
C10—C9—H9A	120.00	O2S—C2S—H2S3	109.00
C8—C9—H9A	120.00	O2S—C2S—H2S1	110.00
C11—C10—H10A	120.00	H2S1—C2S—H2S3	110.00
C9—C10—H10A	120.00	H2S2—C2S—H2S3	109.00
C12—C11—H11A	121.00	H2S1—C2S—H2S2	110.00
			
C4—N1—C1—C2	22.8 (3)	C1A—C2A—C3A—C4A	3.1 (4)
C5—N1—C1—C2	149.4 (3)	O2A—C3A—C4A—Cl2A	−2.3 (4)
C1—N1—C4—C3	0.4 (3)	O2A—C3A—C4A—C5A	175.1 (3)
C5—N1—C4—C3	−123.9 (3)	C2A—C3A—C4A—Cl2A	176.54 (19)
C1—N1—C5—C6	−176.9 (2)	C2A—C3A—C4A—C5A	−6.1 (4)
C4—N1—C5—C6	−54.8 (3)	Cl2A—C4A—C5A—O3A	2.2 (4)
C8—N2—C12—C11	1.3 (4)	Cl2A—C4A—C5A—C6A	−178.9 (2)
C12—N2—C8—C7	179.0 (2)	C3A—C4A—C5A—O3A	−175.2 (3)
C12—N2—C8—C9	−3.0 (4)	C3A—C4A—C5A—C6A	3.7 (4)
N1—C1—C2—C3	−37.7 (4)	O3A—C5A—C6A—O4A	−0.1 (4)
C1—C2—C3—C4	38.2 (4)	O3A—C5A—C6A—C1A	−178.9 (3)
C2—C3—C4—N1	−23.8 (4)	C4A—C5A—C6A—O4A	−179.1 (2)
N1—C5—C6—C7	149.1 (3)	C4A—C5A—C6A—C1A	2.1 (4)
C5—C6—C7—C8	−179.2 (2)	Cl1B—C1B—C2B—O1B	−0.4 (4)
C5—C6—C7—C13	1.9 (5)	Cl1B—C1B—C2B—C3B	−179.48 (18)
C13—C7—C8—C9	−118.6 (3)	C6B—C1B—C2B—O1B	176.6 (3)
C6—C7—C13—C14	−142.1 (3)	C6B—C1B—C2B—C3B	−2.5 (4)
C6—C7—C13—C18	39.7 (4)	Cl1B—C1B—C6B—O4B	−0.4 (4)
C8—C7—C13—C14	39.0 (3)	Cl1B—C1B—C6B—C5B	179.70 (18)
C8—C7—C13—C18	−139.2 (3)	C2B—C1B—C6B—O4B	−177.3 (2)
C6—C7—C8—C9	62.4 (4)	C2B—C1B—C6B—C5B	2.7 (4)
C13—C7—C8—N2	59.3 (3)	O1B—C2B—C3B—O2B	3.9 (3)
C6—C7—C8—N2	−119.7 (3)	O1B—C2B—C3B—C4B	−176.7 (2)
N2—C8—C9—C10	2.6 (4)	C1B—C2B—C3B—O2B	−176.9 (2)
C7—C8—C9—C10	−179.5 (3)	C1B—C2B—C3B—C4B	2.4 (4)
C8—C9—C10—C11	−0.5 (4)	O2B—C3B—C4B—Cl2B	−0.8 (4)
C9—C10—C11—C12	−1.1 (4)	O2B—C3B—C4B—C5B	176.5 (2)
C10—C11—C12—N2	0.8 (4)	C2B—C3B—C4B—Cl2B	179.9 (2)
C7—C13—C14—C15	−179.2 (2)	C2B—C3B—C4B—C5B	−2.8 (4)
C18—C13—C14—C15	−0.9 (4)	Cl2B—C4B—C5B—O3B	1.8 (4)
C7—C13—C18—C17	179.9 (2)	Cl2B—C4B—C5B—C6B	−179.56 (18)
C14—C13—C18—C17	1.6 (4)	C3B—C4B—C5B—O3B	−175.6 (3)
C13—C14—C15—C16	−0.7 (4)	C3B—C4B—C5B—C6B	3.0 (4)
C14—C15—C16—C17	1.4 (4)	O3B—C5B—C6B—O4B	−4.1 (4)
C14—C15—C16—C19	179.7 (3)	O3B—C5B—C6B—C1B	175.8 (2)
C15—C16—C17—C18	−0.7 (4)	C4B—C5B—C6B—O4B	177.2 (2)
C19—C16—C17—C18	−178.9 (3)	C4B—C5B—C6B—C1B	−2.9 (3)
C16—C17—C18—C13	−0.9 (4)	O1C—C1C—C2C—Cl1C	−1.6 (4)
Cl1A—C1A—C2A—O1A	−0.7 (4)	O1C—C1C—C2C—C3C	178.6 (3)
Cl1A—C1A—C2A—C3A	179.5 (2)	C3C^i^—C1C—C2C—Cl1C	178.13 (19)
C6A—C1A—C2A—O1A	−177.9 (3)	C3C^i^—C1C—C2C—C3C	−1.6 (4)
C6A—C1A—C2A—C3A	2.3 (4)	O1C—C1C—C3C^i^—O2C^i^	1.2 (4)
Cl1A—C1A—C6A—O4A	−0.8 (4)	O1C—C1C—C3C^i^—C2C^i^	−178.7 (3)
Cl1A—C1A—C6A—C5A	177.9 (2)	C2C—C1C—C3C^i^—O2C^i^	−178.6 (3)
C2A—C1A—C6A—O4A	176.4 (3)	C2C—C1C—C3C^i^—C2C^i^	1.5 (4)
C2A—C1A—C6A—C5A	−4.9 (4)	Cl1C—C2C—C3C—O2C	1.8 (4)
O1A—C2A—C3A—O2A	2.3 (4)	Cl1C—C2C—C3C—C1C^i^	−178.1 (2)
O1A—C2A—C3A—C4A	−176.7 (3)	C1C—C2C—C3C—O2C	−178.5 (3)
C1A—C2A—C3A—O2A	−178.0 (2)	C1C—C2C—C3C—C1C^i^	1.6 (4)

Symmetry code: (i) −*x*+1, −*y*, −*z*+2.

### Hydrogen-bond geometry (Å, º)

**Table:**

*D*—H···*A*	*D*—H	H···*A*	*D*···*A*	*D*—H···*A*
O4*A*—H4*AA*···O3*A*	0.84	2.15	2.629 (3)	116
O4*A*—H4*AA*···O1*W*^ii^	0.84	1.92	2.672 (3)	148
N1—H1*C*···O3*A*	0.93	1.78	2.699 (3)	167
O2*B*—H2*BA*···O1*B*	0.84	2.19	2.655 (3)	115
O2*B*—H2*BA*···O1*A*^iii^	0.84	2.50	3.012 (3)	121
O2*B*—H2*BA*···O2*A*^iii^	0.84	2.08	2.776 (3)	139
N2—H2*C*···O3*B*	0.88	2.55	2.900 (3)	104
N2—H2*C*···O4*B*	0.88	1.79	2.667 (3)	175
O2*C*—H2*CA*···O1*C*^i^	0.84	2.21	2.680 (4)	116
O1*W*—H1*W*1···O1*S*	0.90 (3)	2.06 (3)	2.882 (3)	152 (4)
O1*W*—H1*W*2···O2*B*	0.82 (5)	2.18 (4)	2.976 (3)	162 (4)
C1—H1*B*···O1*A*^iv^	0.99	2.34	3.286 (5)	160
C3—H3*A*···O1*B*^v^	0.99	2.47	3.138 (5)	124
C4—H4*B*···O2*A*^vi^	0.99	2.37	3.229 (3)	144
C4—H4*B*···O1*B*^v^	0.99	2.34	2.994 (3)	123
C5—H5*B*···O1*B*^v^	0.99	2.44	3.186 (3)	132
C9—H9*A*···Cl2*A*^vi^	0.95	2.82	3.525 (3)	131
C9—H9*A*···O2*A*^vi^	0.95	2.46	3.363 (3)	158
C18—H18*A*···Cl2*B*^ii^	0.95	2.68	3.467 (3)	140
C19—H19*B*···O1*A*^iii^	0.98	2.55	3.102 (4)	116

Symmetry codes: (i) −*x*+1, −*y*, −*z*+2; (ii) *x*+1/2, −*y*+1/2, *z*−1/2; (iii) −*x*+3/2, *y*−1/2, −*z*+1/2; (iv) −*x*+2, −*y*+1, −*z*; (v) *x*−1/2, −*y*+1/2, *z*−1/2; (vi) −*x*+1, −*y*+1, −*z*.

## References

[bb1] Adam, M. S., Parkin, A., Thomas, L. H. & Wilson, C. C. (2010). *CrystEngComm*, **12**, 917–924.

[bb2] Agilent (2011). *CrysAlis PRO* and *CrysAlis RED* Agilent Technologies Ltd, Yarnton, England.

[bb3] Al-Attas, A. S., Habeeb, M. M. & Al-Raimi, D. S. (2009). *J. Mol. Struct.* **928**, 158–170.

[bb4] Cremer, D. & Pople, J. A. (1975). *J. Am. Chem. Soc.* **97**, 1354–1358.

[bb5] Dayananda, A. S., Jasinski, J. P., Golen, J. A., Yathirajan, H. S. & Raju, C. R. (2011). *Acta Cryst.* E**67**, o2502. 10.1107/S160053681103457X PMC3200727 22064274

[bb6] Dutkiewicz, G., Yathirajan, H. S., Al-arique, Q. N. M. H., Narayana, B. & Kubicki, M. (2010). *Acta Cryst.* E**66**, o497–o498. 10.1107/S1600536810003387 PMC2979702 21579900

[bb7] Farrugia, L. J. (1997). *J. Appl. Cryst.* **30**, 565.

[bb8] Farrugia, L. J. (1999). *J. Appl. Cryst.* **32**, 837–838.

[bb9] Gotoh, K., Maruyama, S. & Ishida, H. (2010). *Acta Cryst.* E**66**, o3255. 10.1107/S1600536810047744 PMC3011634 21589539

[bb10] Hakim Al-arique, Q. N. M., Jasinski, J. P., Butcher, R. J., Yathirajan, H. S. & Narayana, B. (2010). *Acta Cryst.* E**66**, o1507–o1508. 10.1107/S1600536810019665 PMC2979375 21579567

[bb11] Jasinski, J. P., Butcher, R. J., Hakim Al-arique, Q. N. M., Yathirajan, H. S. & Narayana, B. (2010). *Acta Cryst.* E**66**, o163–o164. 10.1107/S1600536809053410 PMC2980187 21580051

[bb12] Parvez, M. & Sabir, A. P. (1997). *Acta Cryst.* C**53**, 679–681.

[bb13] Sandhya, B., Hegde, A. H., Kalanur, S. S., Katrahalli, U. & Seetharamappa, J. (2011). *J. Pharm. Biomed. Anal.* **54**, 1180–1186. 10.1016/j.jpba.2010.12.012 21215548

[bb14] Sheldrick, G. M. (2008). *Acta Cryst.* A**64**, 112–122. 10.1107/S0108767307043930 18156677

[bb15] Spek, A. L. (2009). *Acta Cryst.* D**65**, 148–155. 10.1107/S090744490804362X PMC2631630 19171970

[bb16] Udachin, K. A., Zaman, M. B. & Ripmeester, J. A. (2011). *Acta Cryst.* E**67**, o2625. 10.1107/S1600536811036373 PMC3201558 22058767

